# Dental pathologies in lamniform and carcharhiniform sharks with comments on the classification and homology of double tooth pathologies in vertebrates

**DOI:** 10.7717/peerj.12775

**Published:** 2022-05-11

**Authors:** Harrison S. Miller, Haviv M. Avrahami, Lindsay E. Zanno

**Affiliations:** 1 Department of Biological Sciences, North Carolina State University, Raleigh, North Carolina, United States; 2 North Carolina Museum of Natural Sciences, Raleigh, North Carolina, United States

**Keywords:** Shark, Tooth, Double tooth, Chondrichthyan, Otodus megalodon, *Carcharhinus leucas*, Lamniformes, Carcharhiniformes, Dentition, Pathology

## Abstract

Double tooth pathologies are important indicators of trauma, disease, diet, and feeding biomechanics, and are widely documented in mammals. However, diagnosis of double tooth pathologies in extinct non-mammalian vertebrates is complicated by several compounding factors including: a lack of shared terminology reflecting shared etiology, inconsistencies in definitions and key features within and outside of mammals (*e.g*., gemination, fusion, twinning, concrescence); differences in tooth morphology, heterodonty, regeneration, and implantation between mammals and non-mammalian vertebrates; and the unmet need for diagnostic criteria that can be applied to isolated teeth, which are common in the fossil record. Here we report on double tooth pathologies in the lamniform and carcharhiniform Cenozoic sharks *Otodus megalodon* (NCSM 33639) and *Carcharhinus leucas* (NCSM 33640, 33641). All three teeth bear a singular bifid crown with mirrored halves and abnormal internal microstructure—a single, bifurcating pulp cavity in *C. leucas* and a more than tripling of vessels in *O. megalodon* (from two to seven main ascending canals). We identify these abnormalities as likely examples of gemination due to their symmetry, which rules out fusion of tooth buds in one tooth file in different developmental stages in polyphyodont taxa; however, we note that incomplete forms of mesiodistal tooth fusion can be morphologically indistinguishable from gemination, and thus fusion cannot be rejected. We further compile and recategorize, when possible, the diversity of tooth pathologies in sharks. The identification of double tooth pathologies in *O. megalodon* and *C. leucas* has paleobiological implications. Such pathologies in sharks are largely hypothesized to stem from trauma to developing tooth buds. *Carcharhinus leucas* is known to feed on prey documented to cause feeding-related oral traumas (*e.g*., rays, sawfish, spiny fish, and sea urchins). However, *O*. *megalodon*, is considered to have largely fed on marine mammals, and perhaps turtles and/or fish, raising the possibility that the dietary diversity of this species is, as of yet, underappreciated. The genetic underpinnings of tooth morphogenesis and regeneration is highly conserved throughout vertebrate evolution, suggesting a homologous framework can be established. However, more research is needed to link developmental, paleobiological, and/or paleoenvironmental factors to gemination/fusion in polyphyodont taxa. We argue that the definitions and diagnostic criteria for dental pathologies in vertebrates require standardization in order to advance macroevolutionary studies of feeding trauma in deep time.

## Introduction

A wide array of dental pathologies have been reported across multiple vertebrate clades, in both extant (Aalderink et al., 2015; Crossley et al., 1998; Jett et al., 2017; Jones & Franklin, 2006; Scarpetta & Bell, 2020; Shen et al., 2011; Winer et al., 2016b; Winer, Liong & Verstraete, 2013) and extinct taxa (Candeiro & Tanke, 2008; Jäger, Cifelli & Martin, 2020; Kirillova, 2009; Matthias, McWhinney & Carpenter, 2016; Reisz et al., 2011; Xing et al., 2013). In particular, a category of dental pathologies known as tooth doubling, or connate teeth, is well documented in extant mammalian clades, especially humans (Agnihotri, Marwah & Goel, 2007; Camargo, Aritaa & Watanabe, 2016; Cetinbas et al., 2007; Ertaş et al., 2014; Guler et al., 2013; Hülsmann, Bahr & Grohmann, 1997; Hunasgi et al., 2017; Jain, Yeluri & Munshi, 2014; Kamura, 2019; Knežević et al., 2002; Mahendra et al., 2014; Sharma et al., 2015; Shokri, Baharvand & Mortazavi, 2013; Syed et al., 2016; Tasa, 1998; Tsesis et al., 2003).

**Figure 1:**
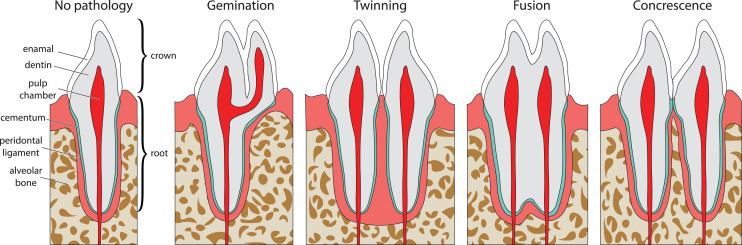
Idealized expressions of double tooth pathologies of stylized mammalian incisor teeth in lingual view. Illustrations of mammalian incisor teeth showing no pathology, gemination, twinning, fusion, and concrescence. Note that there is a spectrum of manifestations of these pathologies and that gemination, fusion, and concrescence can overlap in morphology depending on their stage of development.

Double tooth pathologies occur when either a single tooth splits into two teeth (gemination and twinning; Lucas & Schoch, 1987; Fig. 1A) or when two or more teeth merge (fusion and concrescence; Lucas & Schoch, 1987; Figs. 1B–1D). These pathologies are not mutually exclusive, and although rare, can occur in tandem. Specifically, there are documentations of fusion and concrescence co-occurring in humans (Aldred, Gordon & Talacko, 2011; Syed et al., 2016) and felines (Verstraete et al., 1996).

Gemination (Fig. 1A) is the result of a partial division of a single tooth bud (Lucas & Schoch, 1987). It is the most commonly reported double tooth pathology with a wide representation across terrestrial and marine mammals, including extant representatives of Hominidae (Agnihotri, Marwah & Goel, 2007; Camargo, Aritaa & Watanabe, 2016; Ertaş et al., 2014; Jain, Yeluri & Munshi, 2014; Knežević et al., 2002; Mahendra et al., 2014; Sharma et al., 2015; Shokri, Baharvand & Mortazavi, 2013; Tasa, 1998; Tsesis et al., 2003), Pinnipedia (Abbott & Verstraete, 2005; Kahle et al., 2018), Felidae (Aghashani et al., 2016; Gomerčić et al., 2009; Mestrinho et al., 2018), Ursidae (Clark et al., 2017), Cercopithecidae (Colyer, 1928), Equidae (Easley, 2006), Talpidae (Feldhamer & Towery, 2010; Kawada et al., 2006; Kawada et al., 2011), Canidae (Gisburne & Feldhamer, 2005; Hitchin & Morris, 1966), Mustelidae (Hauer, 2002), Cetacea (Loch et al., 2011; Norton, 2009), and Muridae (Sofaer, 1969). Gemination is also reported in the extinct taxa Coryphodontidae (Lucas & Schoch, 1987), Condylarthra (Rose & Smith, 1979), and *Mammuthus* (Burns, Baker & Mol, 2003).

Twinning (also referred to as schizodontia) (Fig. 1B) is identified as two mirrored teeth occupying a single tooth position and is thought to be caused by the complete cleavage of a single tooth bud (essentially complete gemination of the tooth) (Lucas & Schoch, 1987). It has been reported in Hominidae (Hunasgi et al., 2017; Sharma et al., 2015), Canidae (Fine, 1964), and Condylarthra (Rose & Smith, 1979).

Fusion (also referred to as synodontia) (Fig. 1C) occurs when teeth are united by their dentine and/or enamel due to the complete or incomplete union of two or more tooth buds during development (Lucas & Schoch, 1987). Fusion has been reported in Hominidae (Cetinbas et al., 2007; Guler et al., 2013; Hülsmann, Bahr & Grohmann, 1997; Kamura, 2019; Syed et al., 2016), Pinnipedia (Winer et al., 2016a), and Felidae (Verstraete et al., 1996). However, in some of these cases, a more general definition of fusion is used that includes concrescence, therefore in the literature it can be difficult to discriminate between reports of these conditions (*e.g*., Cetinbas et al., 2007; Hülsmann, Bahr & Grohmann, 1997). In other cases, tooth doubling is reported without discrimination between fusion or gemination, so the exact condition is unclear (Tsesis et al., 2003).

Concrescence (Fig. 1D) occurs when the roots of two or more teeth are united by cementum or dentine after complete morphogenesis (Lucas & Schoch, 1987), whereby the teeth are complete and conjoined. It has been reported in Hominidae (Kamura, 2019; Syed et al., 2016), Pinnipedia (Kryukova, 2017), Talpidae (Asahara, Kryukov & Motokawa, 2011), Muridae (Peterková et al., 2000), Felidae (Verstraete et al., 1996), and Equidae (Spasskaya, 2014).

The etiological factors that contribute to these pathologies are not well known and likely vary across vertebrate clades, but several have been suggested including vitamin deficiency, hormonal irregularities, infection, inflammation of surrounding tissues, genetic predispositions, hereditary or congenital diseases, nutritional deficiency, local traumas, ionizing radiation, endocrine influences, environmental factors, space restriction during development, and excessive occlusal force (Guler et al., 2013; Hunasgi et al., 2017; Mahendra et al., 2014; Syed et al., 2016). These etiological factors have been cited as contributing factors in other tooth pathologies as well. For example, trauma, aberrant tooth replacement, and genetic expression have been proposed to cause split carinae (termed cutting edges in Chondrichthyes) (Welsh, Boyd & Spearing, 2020)—a tooth pathology wherein the serrated cutting surface of the tooth bifurcates abnormally. This pathology is reported in extinct taxa such as theropod dinosaurs (Tyrannosauridae, Erickson, 1995; Paraves, Fiorillo & Gangloff, 2001; Han et al., 2011; and Carcharodontosauridae, Candeiro & Tanke, 2008), mammals (Nimravidae, Welsh, Boyd & Spearing, 2020), and fish (*Otodus megalodon*, Itano, 2013).

Although tooth doubling is widely reported in extinct taxa, the currently accepted subtypes of double tooth pathologies that utilize a developmental framework were categorized based on a mammalian model (Pindborg, 1970). Discriminating among different types of double tooth pathologies is difficult in the absence of developmental data and/or preservation of complete dentition (More & Tailor, 2012; Patil et al., 2013; Camargo, Aritaa & Watanabe, 2016). Given that the majority of pathological fossil shark teeth are recovered as isolated elements (Martínez-Pérez et al., 2018; Becker, Chamberlain & Stoffer, 2000) and that the morphology of the dentition of sharks and mammals differ, refined diagnoses of double tooth pathologies in shark teeth based on developmental history are lacking. Double tooth pathologies including those simply described as bicuspid and/or coalescent teeth have been reported in chondrichthyans (*Leonodus carlsi* (Botella, 2006; Botella, Valenzuela-Ríos & Martínez-Pérez, 2009), Batoidea (Becker, Chamberlain & Stoffer, 2000; Delpiani, Figueroa & Mabragaña, 2012; Romer, 1942), Chlamydoselachidae (Gudger, 1937), Heterodontidae (Gudger, 1937), Carcharhiniformes (Balbino & Antunes, 2007; Becker, Chamberlain & Stoffer, 2000; Gudger, 1937), and Lamniformes (Agassiz, 1843; Balbino & Antunes, 2007; Becker, Chamberlain & Stoffer, 2000; Boessenecker, 2016; Cappetta & Case, 1975; Davis, 1890; Hubbell, 1996; Itano, 2013; Roemer, 1849; Shimada, 1997; Vuuren et al., 2015)). In the absence of a developmental diagnosis, questions remain about the commonality, homology, and phylogenetic distribution of the various types of tooth doubling in the fossil record, as well as the equivalence of these pathologies between mammals and chondrichthyans.

Here we describe double tooth pathologies in the lamniform *Otodus megalodon* and the carcharhiniform *Carcharhinus leucas*, two Cenozoic shark species that vastly differ in ecology and tooth morphology. *C. leucas*, commonly known as the bull shark, evolved during the Miocene (Matich & Heithaus, 2012). It is a widely distributed coastal predator found in tropical, subtropical, and temperate ecosystems and is a highly efficient osmoregulator that can travel between fresh and marine waters and respond to sudden changes in salinity with minimal metabolic costs (Matich & Heithaus, 2012). The maximum body size of *C. leucas* has been reported to be in the range of 2.85–3.27 m (Habegger et al., 2012; Hoarau et al., 2021). Most carcharhiniforms, including *C. leucas*, exhibit the orthodont tooth histotype (*i.e*., they have hollow pulp cavities), the second most common histotype in sharks (Jambura et al., 2020; Moyer, Riccio & Bemis, 2015).

*O. megalodon* was a much larger shark, estimated to reach maximum body sizes in the range of 14.2–18 m (Pimiento & Balk, 2015; Shimada, 2019) and was a globally distributed apex predator of marine ecosystems for as many as 14 million years. *O. megalodon* appears in the fossil record around the middle Miocene (15.9 Ma) (Pimiento & Clements, 2014), but there are varying opinions on when it went extinct. Some suggest that *O. megalodon* went extinct near the Pliocene/Pleistocene boundary (2.6 Ma) (Pimiento & Clements, 2014), whereas others propose a much earlier extinction around the end of the early Pliocene (3.6 Ma) suggesting that specimens found in a locality dated later than this have been reworked (Boessenecker et al., 2019). Lamniforms, such as *O*. *megalodon* exhibit osteodont dentition (their pulp cavities are filled with osteodentine), a histotype so far known to be exclusive to this group (Jambura et al., 2020; Moyer, Riccio & Bemis, 2015).

In order to determine whether gemination, fusion, twinning, concrescence, or some combination can be substantiated in these teeth, we describe their gross and internal morphology using nano-CT imaging. We then examine the morphological evidence for the formation of each pathological tooth. Identifying the types of tooth pathologies and their distribution among vertebrate clades can provide important paleobiological information on tooth developmental anomalies, and injuries, with potential implications for behavior, such as feeding traumas in selachians.

## Materials and Methods

### Specimens

Our figured specimen sample consists of six Cenozoic shark teeth, representing pathological and non-pathological examples of two species–*Otodus megalodon* and *Carcharhinus leucas*. We examined three *C. leucas* teeth, two with a double tooth pathology (NCSM 33640 and NCSM 33641) and four non-pathological examples (NCSM 34038) as well as >700 non-pathological *Carcharhinus* sp. teeth from the NCSM collections. For *O. megalodon*, we studied one pathological tooth (NCSM 33639), two non-pathological teeth (NCSM 9545 and NCSM 14984), and examined over >200 non-pathological *O. megalodon* teeth from the NCSM collections. NCSM 33640 and NCSM 33641 were collected at Venice Beach, Sarasota County, Florida. NCSM 34038 and NCSM 9545 were collected from the Pliocene Yorktown Formation. NCSM 33639 was collected 72.42 km off the coast of Wrightsville Beach, New Hanover County, North Carolina. NCSM 14984 was collected from the Pliocene Bear Bluff Formation at an annex off of SR-1700, 2.59 km south of the center of Elizabethtown, Bladen County, North Carolina.

### Taxonomy and terminology

Here we follow Jambura et al. (2019) and Shimada (2019) in placing *O. megalodon* within the genus *Otodus* and subclade Otodontidae. Some authors propose alternative genus-species combinations, such as *Carcharodon megalodon* or *Carcharocles megalodon* (*e.g*. Cappetta, 1987; Purdy et al., 2001; Pimiento, 2010; Boessenecker, 2016). This ongoing taxonomic debate does not affect our results. We follow Cappetta (2012) and Shimada (2002) for dental terminology including the labial (external) and lingual (internal) face, distal (further from the mid-point of the jaw) and mesial side (closer to the midpoint of the jaw), anterior (closer to the front of the jaw) and posterior (closer to the back of the jaw) position, root (anchors the tooth to the jaw), neck (borderline between root and crown), crown (cap composed of dentine and enameloid that is attached to the root), cusp (sharp point formed by the tip of the crown), apex (tip of the crown), cutting edge (smooth or serrated edge of the crown), serrations (individual sharp points along the cutting edge), basal ledge (distinct ledge seen on the labial side at the base of the crown), central foramen (distinct vascular opening on the lingual side of the tooth), nutrient groove (vascular groove in the root leading to central foramen). We follow Martínez-Pérez et al. (2018) and Ivanov & Nilov (2016) for vascular terminology (*e.g*., main ascending, secondary ascending, secondary horizontal canals, small horizontal, small vertical (or ascending), large longitudinal (or semicircular), and small branching secondary canals) and Pindborg (1970) for the definitions of double tooth pathology types.

### Gross morphological data

**Figure 2:**
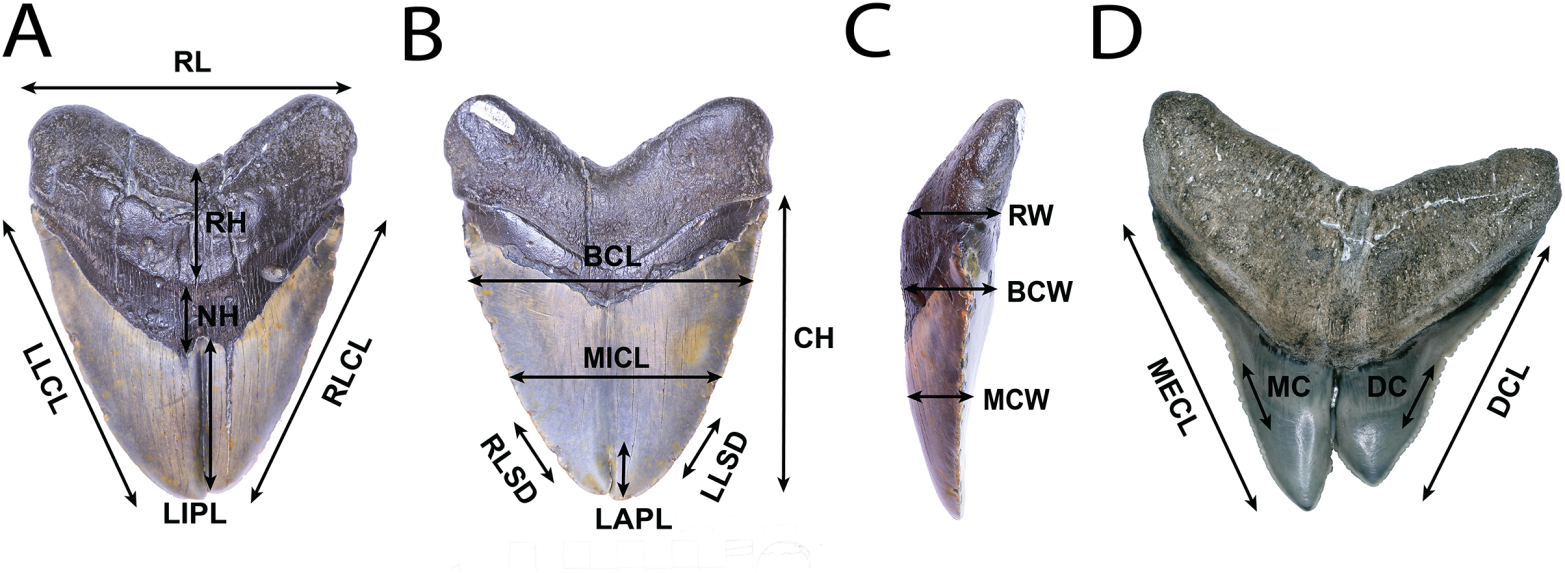
Anatomical abbreviations. All linear measurements used for analysis of the external morphology of the specimens. (A) (NSCM 33639: *O. megalodon*; lingual view) NH, Neck Height; RH, Root Height; RL, Root Length; LIPL, Lingual Pathology Length; LLCL, Left Lateral Crown Length in Lingual View; and RLCL, Right Lateral Crown Length in Lingual View. (B) (NCSM 33639; *O. megalodon* labial view) CH, Crown Height; MICL, Mid-Crown Length; BCL, Basal Crown Length; and LAPL, Labial Pathology Length; LLSD, Left Lateral Serration Density; and RLSD, Right Lateral Serration Density. (C) (NCSM 33639; *O. megalodon*; lateral view) MCW, Mid-Crown Width; BCW, Basal Crown Width; and RW, Root Width. (D) (NCSM 33640; *C. leucas*; lingual view) MECL, Mesial Crown Length; DCL, Distal Crown Length; MC, Mesiocentral Serration Density; and DC, Distocentral Serration Density.

All teeth were photographed using either a DSLR camera, in conjunction with image stacking operations in Adobe Photoshop, or a Keyence VHX-1000E image stacking microscope. Linear, angular, and serration density measurements were collected using digital calipers, or a Keyence VHX-1000E image stacking microscope and ImageJ 1.53e (Rasband & Image, 2011). Measurement standards are illustrated in Fig. 2 and include: Crown Height (CH), Mesial Crown Length (MECL), Distal Crown Length (DCL), Mid-Crown Length (MICL), Basal Crown Length (BCL), Mid-Crown Width (MCW), Basal Crown Width (BCW), Neck Height (NH), Root Height (RH), Root Length (RL), Root Width (RW), Labial Pathology Length (LAPL), Lingual Pathology Length (LIPL), Mesiocentral Serration Density (MC), Distocentral Serration Density (SC), Left Lateral Crown Length in Lingual View (LLCL), Right Lateral Crown Length in Lingual View (RLCL), Left Lateral Serration Density in Lingual View (LLSD), and Right Lateral Serration Density in Lingual View (RLSD). CH, MECL, and DCL were modified from Whitenack & Motta (2010), MICL, BCL, MCW, BCW, MC, and DC were modified from Hendrickx, Mateus & Araújo (2015), and NH, RH, RL, RW. LAPL, LIPL, LLCL, RLCL, LLDD, and RLDD were created for this study. Mesiocentral, distocentral, left lateral, and right lateral serration density measurements were measured along the cutting edge where visible, then mean and variance was taken.

### Nano-CT imaging and segmentation

The internal morphology and structure of the *C. leucas* teeth NCSM 33640, NCSM 33641, and NCSM 34038 and the *O. megalodon* teeth NCSM 9545 and NCSM 33639 were investigated using the ZEISS Xradia 510 Versa X-ray microscope located in the Analytical Instrumentation Facility at North Carolina State University using the following scanning protocol, 160 kV and 63 µA. The CT images were captured using a voxel size of 26.92 μm and image dimensions of 1,024 px × 1,004 px. CT data was segmented by hand without the use of algorithms, and 3D models were produced using the software Avizo Lite 9.0 (Thermo Fisher Scientific, 2018). All micro-CT data and 3D models produced for this study are available in the MorphoSource repository, under project P367889 at https://www.morphosource.org.

## Results

### Otodus megalodon

**Figure 3:**
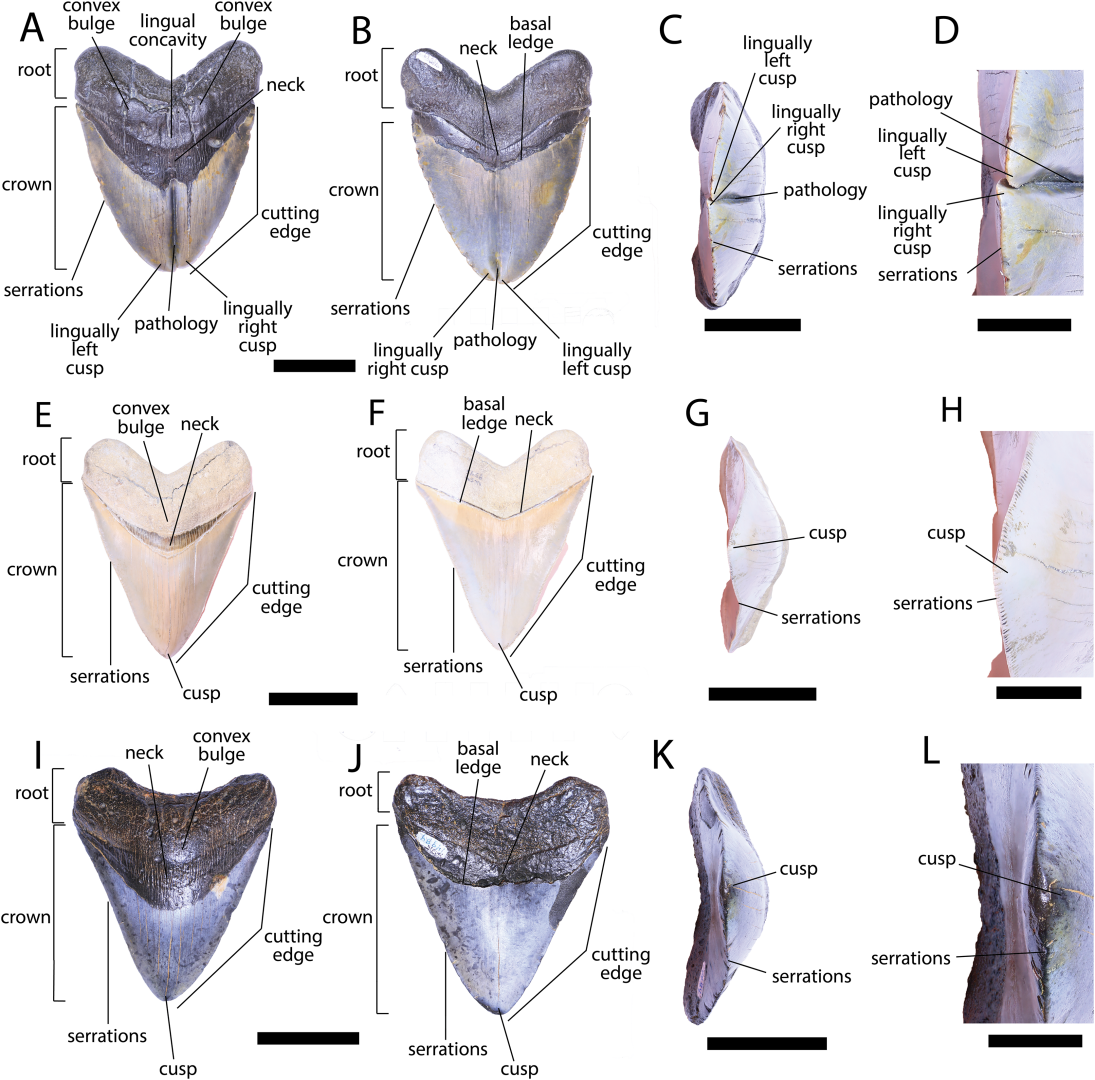
External morphology of *Otodus megalodon* teeth. Pathological *O. megalodon* tooth NCSM 33639 in (A) lingual, (B) labial, and (C) occlusal views. (D) Enlarged view of the pathology and serrations. Non-pathological *O. megalodon* tooth NCSM 9545 in (E) lingual, (F) labial, and (G) occlusal views. (H) Enlarged view of the lack of pathology and serrations. Non-pathological *O. megalodon* tooth NCSM 14984 in (I) lingual, (J) labial, and (K) occlusal views. (L) Enlarged view of the normal apex and serrations of non-pathological *O. megalodon* tooth NCSM 14984. Scale bar equals 5 cm for views A–C, E–G, & I–K and 1 cm for view D, H, & L.

We compiled the diagnostic characteristics previously published in Purdy et al. (2001), Pimiento (2010), and Boessenecker (2016) to refer NCSM 9545, 14984, and 33639 to the otodontid (megatoothed) shark *O. megalodon*. Our referral is based on: large size (crown height 107.59–91.10 mm, Table 1); a large chevron-shaped neck bearing thin enameloid (Pimiento, 2010; Boessenecker, 2016); fine serrations (0.75–1.47 serrations per mm on left lateral cutting edge in lingual view; Table 1) (Pimiento, 2010; Boessenecker, 2016); a convex lingual face (Pimiento, 2010), a slightly convex to flat labial face (Pimiento, 2010), and absence of cusplets on large teeth (Purdy et al., 2001). All teeth are highly symmetrical (Fig. 3) suggesting they represent anterior teeth (Purdy et al., 2001), this combined with the large, broad nature of the teeth suggests they were located in the upper jaw (Smith et al., 2018). However, due to the highly symmetrical nature of the tooth, we are unable to determine if the tooth derives from the left or right side of the upper jaw, thus we use the terms “left lateral/right lateral” in place of “mesial/distal.”

**Table 1:** Anatomical measurements.

Specimen number	NCSM 33639	NCSM 9545	NCSM 14984	NCSM 34038	NCSM 33641	NCSM 33640
Species	*O. megalodon*	*O. megalodon*	*O. megalodon*	*C. leucas*	*C. leucas*	*C. leucas*
Crown Height (CH)	107.59	99.34	91.10	15.12	14.06	13.78
Mesial Crown Length (MECL)	–	–	–	16.89	17.18	16.25
Distal Crown Length (DCL)	–	–	–	13.16	15.21	15.00
Left Lateral Crown Length in Lingual View (LLCLL)	120.22	110.30	97.77	–	–	–
Right Lateral Crown Length in Lingual View (RLCLL)	114.70	106.12	104.76	–	–	–
Mid-Crown Length (MICL)	76.42	55.17	50.42	6.44	4.99	9.21
Basal Crown Length (BCL)	108.37	94.66	97.32	15.10	17.51	18.53
Mid-Crown Width (MCW)	18.10	20.96	14.17	2.32	1.52	2.34
Basal Crown Width (BCW)	30.74	15.67	23.60	4.09	4.10	3.09
Neck Height (NH)	26.78	12.63	26.60	1.12	1.90	1.04
Root Height (RH)	41.58	33.42	28.98	6.28	5.81	8.35
Root Length (RL)	113.95	94.70	97.89	15.63	19.86	19.86
Root Width (RW)	29.44	23.98	20.77	3.85	4.33	4.85
Lingual Pathology Length (LIPL)	62.62	0.00	0.00	0.00	5.11	7.51
Labial Pathology Length (LAPL)	13.31	0.00	0.00	0.00	2.98	5.40
Average Mesiocentral Serration Density per mm (MC)	–	–	–	3.41	3.01	3.38
Average Distocentral Serration Density per mm (DC)	–	–	–	3.39	3.24	2.66
Average Left Lateral Serration Density in Lingual View per mm (LLSD)	1.45	1.47	0.75	–	–	–
Average Right Lateral Serration Density in Lingual View per mm (RLSD)	1.76	1.59	1.03	–	–	–
Mesiocentral Serration Density per mm (MC) Variance	–	–	–	1.93	0.29	0.18
Distocentral Serration Density per mm (DC) Variance	–	–	–	2.43	0.02	0.48
Left Lateral Serration Density in Lingual View per mm (LLSD) Variance	0.03	0.01	0.02	–	–	–
Right Lateral Serration Density in Lingual View per mm (RLSD) Variance	0.06	0.02	0.16	–	–	–

**Note:**

Table shows the value measured for each anatomical feature pertaining to the six teeth in the study. All measurements are in cm unless otherwise specified in the table.

The pathological *O. megalodon* tooth, NCSM 33639 (Fig. 3) is the largest in our sample with a crown height of 107.59 mm and a basal crown length of 108.37 mm. Cutting edges are dull and abraded, but where preserved, the mean value of individual serrations is 1.45 per mm on the left lateral cutting edge in lingual view and 1.76 per mm on the right lateral cutting edge in lingual view (Table 1; Fig. 3D). In lingual view, the crown is split medially from the apex to the neck forming two discrete cusps (Fig. 3A). Whereas in labial view, the division appears incompletely developed, expressed as a shallower groove and restricted to the crown tip (Fig. 3B). The tip of the left lateral cusp in lingual view is slightly more extensive and overlaps the right lateral cusp in lingual view (Fig. 3D). The left lateral cusp in lingual view is slightly taller and mesiodistally longer than the right lateral cusp in lingual view. The basal ledge is fairly pronounced compared to other *O. megalodon* teeth in our study sample.

In our sample of both pathological and non-pathological *O. megalodon* teeth the labial surface of the root is broadly flat or concave slightly, and the lingual surface is broadly convex. On the non-pathological *O. megalodon* teeth (NCSM 9545 and 14984) there is a convex bulge that occurs across the lingual surface of the root, immediately basal to the neck. This bulge is a prominent feature in the majority of the *O. megalodon* teeth we examined from the NCSM collections (*n* = 210), although it is reduced or absent in some examples (*e.g.*, NCSM 8759 has a flattened surface and NCSM 32010 is slightly concave in this area). However, on the pathological *O. megalodon* tooth (NCSM 33639) there is a distinct concavity in this region bordered mesially and distally by a subtle bulge (Figs. 3A, 3C).

**Figure 4:**
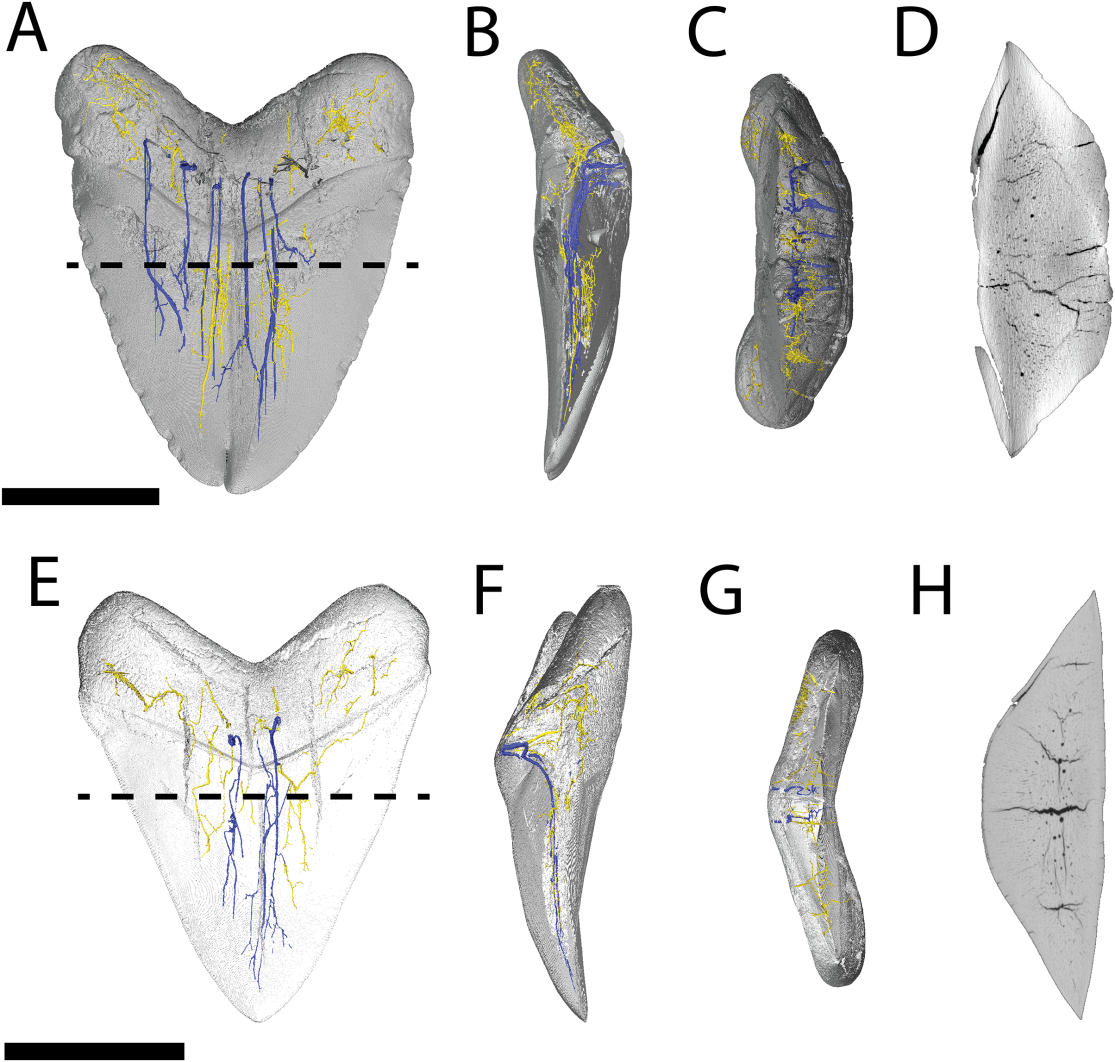
Internal morphology of *Otodus megalodon* teeth. 3-D model (A–C) and nano-CT scan slice (D) of pathological *O. megalodon* tooth NCSM 33639 showing internal structures, primarily the lack of a pulp cavity, six ascending canals (highlighted in blue), and secondary canals (highlighted in yellow) in (A) labiolingual, (B) mesiodistal, and (C and D) occlusal views. 3-D model (E–G) and Nano-CT scan slice (H) of non-pathological *O. megalodon* tooth NCSM 9545 showing internal structures, primarily the lack of a pulp cavity, three ascending canals (blue), and secondary canals (yellow) in (E) labiolingual, (F) mesiodistal, and (G and H) occlusal views. Scale bar equals 5 cm for views A–H. NCSM 14984 not depicted due to COVID-19 restrictions not allowing for Nano-CT scanning. The dashed line on A and E corresponds to where the slices shown in D and H were taken, respectively.

CT data confirms that in pathological (NCSM 33639) and non-pathological (NCSM 9545) examples of *O. megalodon* teeth, the tooth is filled with osteodentine and lacks a pulp cavity as in other selachians with osteodont dentition (Jambura et al., 2018; Jambura et al., 2019) (Fig. 4). Previous scans of *O. megalodon* teeth were noted as not having sufficient resolution (30 µm) to detect the peripheral vascular structure (Jambura et al., 2019). From our scans (<0.7 μm), we were able to visualize most of the vascular network of these *O. megalodon* teeth.

The internal vascular structure of the *O. megalodon* teeth NCSM 9545 and 33639 is similar to two of the vascular networks described by Ivanov & Nilov (2016) (Vascular Systems 2 and 3). Overall, in both teeth, there is a network of small, secondary canals with large, main ascending canals. The small, secondary canals can be further parsed into four types (*sensu* Ivanov & Nilov, 2016): small horizontal, small vertical (or ascending), large longitudinal (or semicircular), and small branching secondary canals. Ivanov & Nilov (2016) use this terminology to describe the vascular systems of orthodont shark teeth, which they note as being more diverse with regard to vascular morphology than osteodont shark teeth. However, Martínez-Pérez et al. (2018) applied these terms to osteodont shark teeth, suggesting that they are broadly applicable.

In both our *O. megalodon* teeth, the main ascending canals extend from the root to the apex of the crown and have a distinct “L” shape in which the canal starts to hook towards the lingual face once it approaches the root (Figs. 4B and 4F), as described in Martínez-Pérez et al. (2018). This is similar to an Early Devonian shark *Leonodus carlsi* tooth documented by Martínez-Pérez et al. (2018) that exhibits main ascendant vascular canals connected at the base of the tooth by a “T”-shape junction and emerging at the base of the labial and lingual regions. Main ascending canals are also roughly twice the diameter of all secondary canals. The average diameter of the main ascending and secondary canals of NCSM 9545 are 369.54 and 184.85 μm, respectively. Whereas, the average diameter of the main ascending and secondary canals of NCSM 9545 are 347.36 and 166.57 μm, respectively (Table S1). The longitudinal canals in both teeth are larger than the other secondary canals, with diameters approximating those of the main ascending canals, but have a semicircular shape and are constrained to the root. Horizontal and vertical secondary canals have similar diameters to branching secondary canals but are longer and more linear.

The pathological *O. megalodon tooth* (NSCM 33639) differs from the non-pathological tooth (NCSM 9545) (Fig. 4A) in that the former has more than double the quantity of main ascending canals (seven) than the latter, which has only two (Fig. 4E). One of these main ascending canals bifurcates toward the apex of the crown in concordance with the externally divided crown and may have been affected by the pathology; there is no bifurcation to this extent seen in NCSM 9545 (Fig. 4). The main ascending canals in NCSM are more concentrated on one side of the tooth as opposed to NCSM 9545 where they are concentrated in the middle. There is also more differentiation in the size of the canals in NCSM 33639; with main ascending canals ranging in diameter from 215.36–753.76 μm and secondary canals ranging from 107.68–269.20 μm, *vs* NCSM 9545; with main ascending canals ranging in diameter from 242.28–511.48 μm and secondary canals ranging from 134.60–269.20 μm (Table S1).

### Carcharhinus leucas

**Figure 5:**
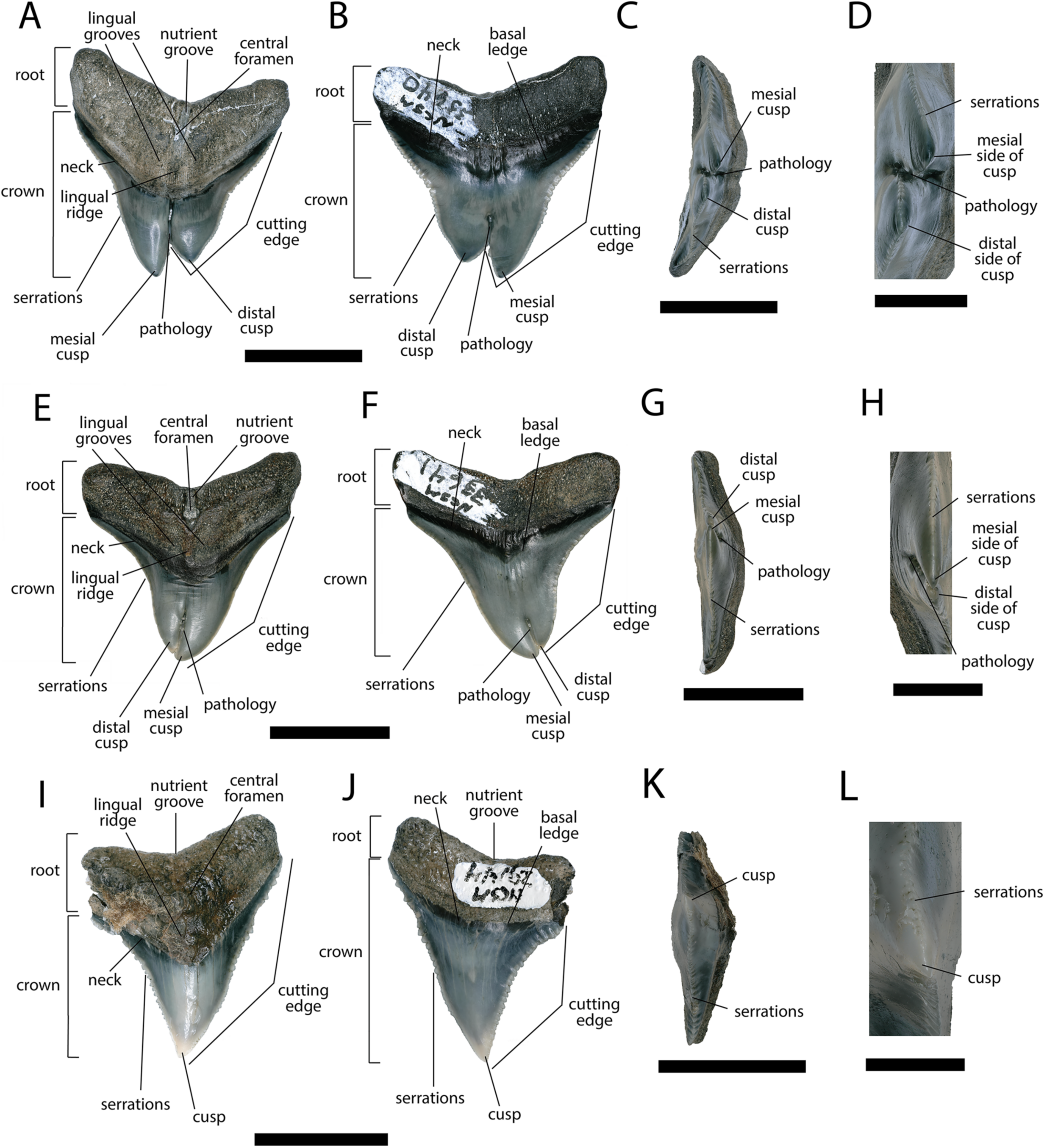
External morphology of *Carcharhinus leucas* teeth. Pathological *C. leucas* tooth NCSM 33640 in (A) lingual, (B) labial, and (C) occlusal views. (D) Enlarged view of the pathology and serrations. Pathological *C. leucas* tooth NCSM 33641 in (E) lingual, (F) labial, and (G) occlusal views. (H) Enlarged view of the pathology and serrations. Non-pathological *C. leucas* tooth NCSM 34038 in (I) lingual, (J) labial, and (K) occlusal views. (L) Enlarged view of the normal apex and serrations. For views A, F, and J, the mesial side of the tooth is on the left and the distal is on the right, it is the inverse for views B, E, and I. Scale bar equals 1 cm for views A–C, E–G, and I–K and 0.5 cm for views D, H, and L.

We compiled the diagnostic characteristics previously published in Purdy et al. (2001) and Marsili (2006) to refer NCSM 34038, NCSM 33640, and NCSM 33641 to the carcharhinid shark *C. leucas*. Our referral is based on: broad, triangular-shaped cusps on upper teeth and arrow-shaped cusps on lower teeth with serrated lateral cutting edges (Marsili, 2006; Fig. 5); coarser serration near the base than the apex of the crown (Marsili, 2006; Fig. 5); straight or slightly wavy mesial cutting edge that is sometimes weakly convex near the tip of the cusp (Purdy et al., 2001; Fig. 5); concave distal cutting edge (Marsili, 2006; Fig. 5); convex lingual face of the crown characterized by a well-developed neck-area (Marsili, 2006; Figs. 5B, 5F, 5J); flat labial face of crown (Marsili, 2006; Figs. 5A, 5E, 5I); and high root characterized by a lingual nutrient groove (Marsili, 2006; Fig. 5). NCSM 34038 and NCSM 33641 are broad and serrated, suggesting they are anterior teeth from the upper jaw (Smith et al., 2018). NCSM 33640 is long, slender, and dull, suggesting it is an anterior tooth from the lower jaw (Smith et al., 2018).

The pathological *C. leucas* tooth, NCSM 33640, exhibits cutting edges that are sharp and well preserved. Where preserved, the mean value of individual serrations is 3.38 per mm on the mesial cutting edge and 2.66 on the distal cutting edge (Table 1; Fig. 5D). In lingual view, the crown is split medially from the apex to the neck forming two discrete cusps (Fig. 5A). Whereas in labial view, the division appears incompletely developed, expressed as a shallower groove and restricted to the midpoint of the crown (Fig. 5B). In addition, the two cusps have been separated mesiodistally, this is the only pathological tooth in the our sample to exhibit this separation. Due to this separation, the inside edges of each side of the cusp bear serrations (Fig. 5D). The mesial cusp is taller and mesiodistally shorter than the distal cusp.

The pathological *C. leucas* tooth, NCSM 33641, exhibits cutting edges that are fairly dull. Where preserved, the mean value of individual serrations is 3.01 mm on the mesial cutting edge and 3.24 mm on the distal cutting edge (Table 1; Fig. 5H). In lingual view, the crown is split medially from the apex to the neck forming two discrete cusps (Fig. 5E). Whereas in labial view, the division appears incompletely developed, expressed as a shallower groove and restricted to the crown tip (Fig. 5F). The mesial cusp is taller and mesiodistally longer than the distal cusp. Both the mesial and distal cusp exhibit curvature. However, the distal cusp exhibits mesially directed curvature, whereas the mesial cusp exhibits distally directed curvature; similar to the rest of the crown, and folds underneath the distal side.

In our sample of non-pathological *C. leucas* teeth NCSM 34038 (*n* = 1) and NCSM 29144 (*n* = 4), each tooth, except a single poorly preserved tooth, exhibits a fully or partially preserved nutrient groove. When well-preserved, the nutrient groove begins at the base of the root along the midline and travels apically halfway up the root before merging with the central foramen. In the largest and best preserved *C. leucas* tooth from the NCSM 29144 lot the nutrient groove continues apically from the central foramen as a single shallow fossa before terminating at the crown enameloid. This single apically directed midline fossa is also present in well-preserved teeth in the NCSM collections previously identified only to the genus level as *Carcharhinus* sp. (*n* > 700). In both pathological *C. leucas* teeth this midline lingual groove is absent, and instead there is an apically oriented midline ridge, which is paralleled mesially and distally by two subtle grooves. The ridge is pronounced in NCSM 33647 and the grooves are subtle, whereas in NCSM 33640 the ridge is more faint and the grooves are more prominent.

In our sample (including pathological and non-pathological teeth) CT-scans reveal that, along with the central foramen, smaller vascular canals open across the external surface of the root and within the nutrient groove. Internally, the diameter of the central foramen is distinctly larger than these smaller canals. In the non-pathological tooth NCSM 34038 the central foramen continues as a single canal, traveling apically to open into the pulp cavity. In pathological tooth NCSM 33640 there is a single large central foramen that is offset towards the mesial side of the nutrient groove. Internally, this foramen appears to bifurcate prior to merging with the pulp cavity. Additionally, there are a series of smaller accessory foramina visible across the external surface of the nutrient groove, one of which is offset to the distal side and may represent a second central foramen. However, internally this canal does not appear to maintain a size diameter consistent with the distinctly larger mesially offset central foramen. It is unclear from the external surface or internal scans if the other pathological tooth NCSM 33641 houses multiple central foramen or an internally bifurcating central foramen. Nonetheless, the concavity that houses the central foramen within the nutrient groove appears slightly mesiodistally expanded.

**Figure 6:**
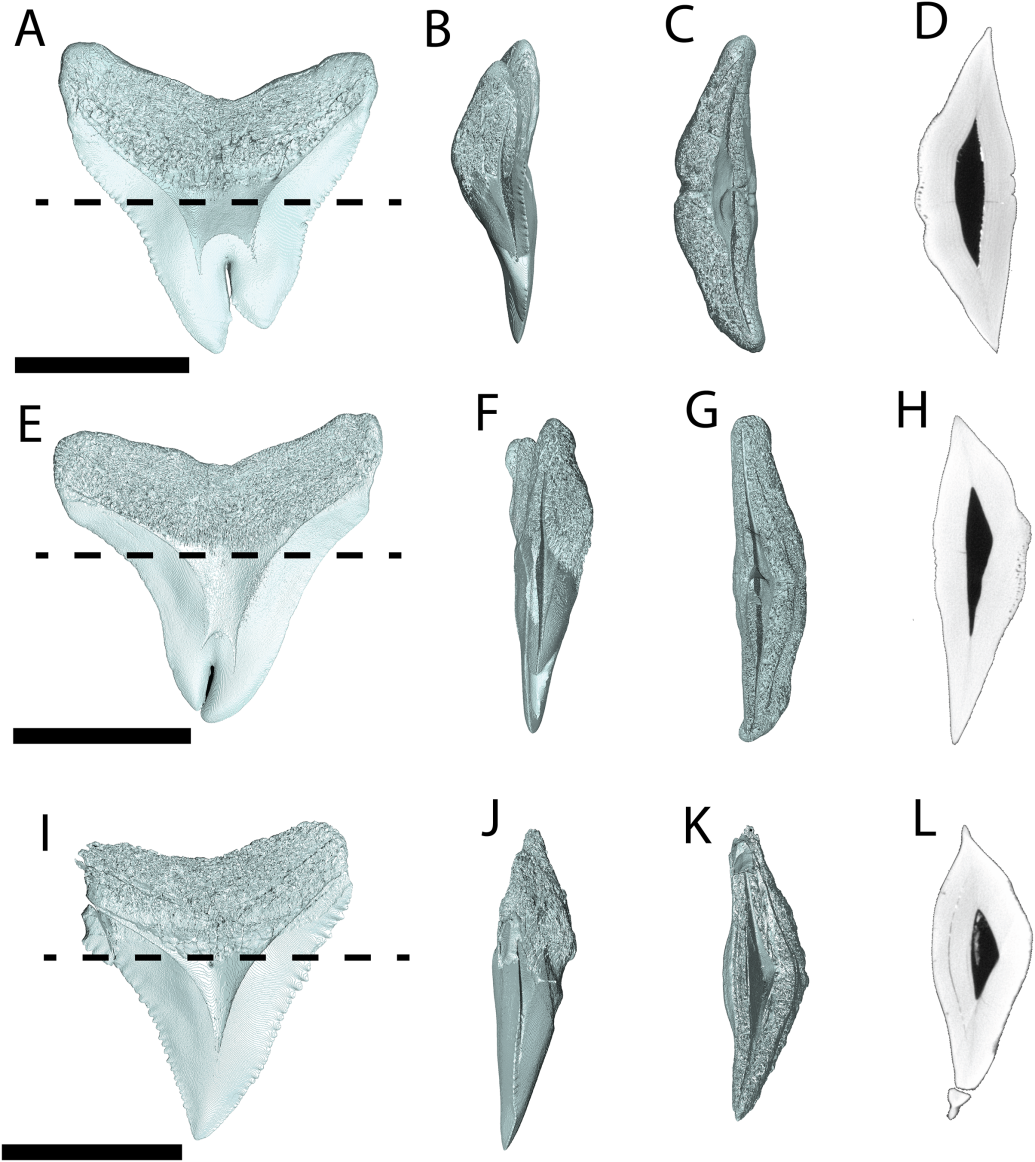
Internal morphology of *Carcharhinus leucas* teeth. 3-D model (A–C) and Nano-CT scan slice (D) of pathological *C. leucas* tooth NCSM 33640 showing internal structures, primarily the presence of a singular, bifurcating pulp cavity in (A) labiolingual, (B) mesiodistal, and (C and D) occlusal views. (E–G) (NCSM 33641) 3-D model (E–G) and Nano-CT scan slice (H) of pathological *C. leucas* tooth NCSM 33641 showing internal structures, primarily the presence of a singular, bifurcating pulp cavity in (E) labiolingual, (F) mesiodistal, and (G and H) occlusal views. A total of 3-D model (I–K) and Nano-CT scan slice (L) of non-pathological *C. leucas* tooth NCSM 34038 showing internal structures, primarily the presence of a singular pulp cavity in (I) labiolingual, (J) mesiodistal, and (K and L) occlusal views. Scale bar equals 1 cm for views A–L. The dashed lines on A, E, and I correspond to where the slices shown in D, H, and L were taken, respectively.

Pathological (NCSM 33640 and NCSM 33641) and non-pathological (NCSM 34038) *C. leucas* teeth possess hollow pulp cavities (Fig. 6). The non-pathological tooth exhibits a single, hollow pulp cavity as documented previously (Jambura et al., 2018). This contrasts with the two pathological teeth examined, which house a bifurcated, single, hollow pulp cavity.

### Variation in pathological teeth

Within our sample of double tooth pathologies in these two species of selachians, we note differences in the extent of crown splitting, bilateral height and recurvature of the doubled apices. For example, whereas the length of the lingual pathology is consistent among all teeth in our sample (extending from the apex of the crown to the neck of the tooth), the pathology length of the labial aspect varies. Crown splitting is restricted to the crown tip on NCSM 33639 (*O. megalodon)* and NCSM 33641 (*C. leucas*), but is restricted to the midpoint of the crown on NCSM 33640 (*C. leucas*). The height of the split apices also varies with the left lateral side of the cusp being taller on NCSM 33639 (*O. megalodon*) and NCSM 33640 (*C. leucas*), and the right lateral side being taller in NCSM 33641 (*C. leucas*), when viewed lingually. Finally, the degree of separation and degree of recurvature between the split crown apices is variable among our sample. Both apices are tightly appressed on NCSM 33639 (*O. megalodon*). The right lateral aspect of NCSM 33641 (*C. leucas*) folds underneath the left lateral aspect in lingual view, whereas the left lateral apex twists over the right lateral apex in lingual view on NCSM 33639. In contrast, the double apices are widely separated on NCSM 33640 (*C. leucas*). These differences are likely idiosyncratic features related to differential timing of gemination or fusion during development.

## Discussion

### Classification and homology of double tooth pathologies

**Figure 7:**
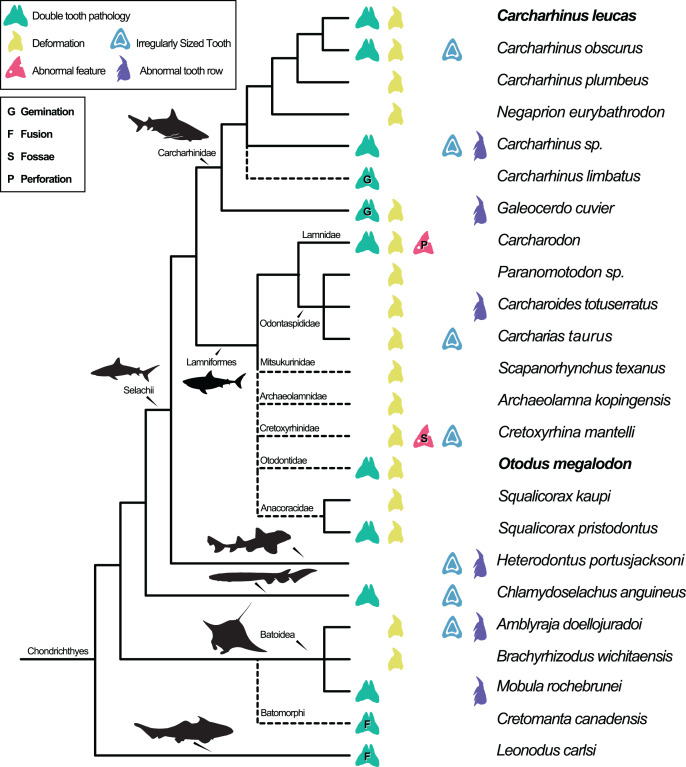
Simplified Chondrichthyes composite phylogeny highlighting the published distribution of dental pathologies. Main branching structure of selachians including the pattern of divergence among Chlamydoselachidae, Heterodontidae, Carcharhinidae, Mitsukurinidae, Odontospididae, the relationships among Carcharhiniformes, and the placement of *Carcharodon* in Lamnidae follows Vélez-Zuazo & Agnarsson (2011). Extinct taxa from Otodontidae, Cretoxyrhinidae, Anacoracidae, and Archaeolamnidae were not included in the molecular analyses that form the backbone of this phylogeny. We have therefore grafted them unresolved within Lamniformes but outside Lamnidae (following Shimada et al., 2017; Siverson & Lindgren, 2005; Rozefelds, 1993; respectively and Cappetta, 2012) relative to the relationships of Mitsukurinidae, Odontospididae, and Lamnidae hypothesized by Vélez-Zuazo & Agnarsson (2011) and Sorenson, Santini & Alfaro (2014). *Cretomanta* is placed unresolved within Batoidae following Underwood & Cumbaa, 2010. *Leonodus* is hypothesized to belong to a clade representing a sister group to all other chondrichthyans (Antarctilamna-Wellerous; Ginter, 2004). Batoidea silhouette adapted from art by Piotr Siedlecki from FreeIMG: https://www.freeimg.net/photo/1471979/manta-ray-sting-silhouette. Selachii silhouettes adapted from art by Faceone911 Glass on toppng: https://toppng.com/free-image/shark-silhouette-PNG-free-PNG-Images_49350. Carcharhinidae and *Heterodontus* silhouettes adapted from art by Francois Libert and John Turnbull, respectively. Lamniformes silhouette from wikimedia commons. *Chlamydoselachus* silhouette adapted from art by Tambja on wikimedia commons. *Leonodus* based on Antarctilamna art by DiBgd from Wikimedia Commons. Silhouettes representing tooth deformation and abnormal tooth row from Becker, Chamberlain & Stoffer (2000) and Gudger (1937). All Silhouettes fall under creative commons fair use. Image sources: [Blacktip Reef Shark, female - *Carcharhinus melanopterus*] (https://www.flickr.com/photos/zsispeo/36123502541), [CC BY-NC-SA 2.0] (https://creativecommons.org/licenses/by-nc-sa/2.0/)- [Port Jackson shark (juvenile) - *Heterodontus portusjacksoni*] (https://www.flickr.com/photos/johnwturnbull/15026942705), [CC BY-NC-SA 2.0] (https://creativecommons.org/licenses/by-nc-sa/2.0/)- [File:Megalodon-Carcharodon-Scale-Chart-SVG] (https://commons.wikimedia.org/wiki/File:Megalodon-Carcharodon-Scale-Chart-SVG.svg), [CC BY-SA 4.0] (https://creativecommons.org/licenses/by-sa/4.0/deed.en)- [File:Chlamydoselachus ang.JPG] (https://commons.wikimedia.org/wiki/File:Chlamydoselachus_ang.JPG), [CC BY-SA 3.0] (https://creativecommons.org/licenses/by-sa/3.0/deed.en)- [File:Antarctilamna speciesDB15.jpg] (https://commons.wikimedia.org/wiki/File:Antarctilamna_speciesDB15.jpg), [CC BY-SA 4.0] (https://creativecommons.org/licenses/by-sa/4.0/deed.en).

Dental pathologies in extant and extinct chondrichthyans are well documented and include a range of abnormalities over a wide phylogenetic distribution (Fig. 7). These deformations include, but are not limited to, notched, split, and deformed cutting edges, cracking of enameloid, excessive dentine growth, deformed tooth crowns, and the development of fossae and perforations (*e.g*., Hubbell, 1996; Shimada, 1997; Becker, Chamberlain & Stoffer, 2000; Itano, 2013; Boessenecker, 2016). Specifically, among Carcharhiniformes and Lamniformes, deformation/rotation/bending/twisting of the tooth, cutting edge deformation, root deformation, irregularly sized teeth, and irregular tooth rows have been reported. Some of the pathologies are only documented in a single genus/species including tooth perforation (*Carcharodon*, Hubbell, 1996), nutrient groove deformation (*Cretoxyrhina mantelli*, Shimada, 1997), and neck deformation (*Cretoxyrhina mantelli*, Shimada, 1997).

Double tooth pathologies are also commonly reported in extant and extinct chondrichthyans. For example, basally conjoined teeth of the Lower Devonian early-diverging chondrichthyan *Leonodus carlsi* (Botella, 2006; Botella, Valenzuela-Ríos & Martínez-Pérez, 2009) are proposed to represent an example of fusion. Whereas, “bicuspid” and/or “coalescent” teeth and/or indeterminate double tooth abnormalities are identified in the Carcharhiniform *Galeocerdo cuvier* (Gudger, 1937, the author; however, uses the junior synonym *Galeocerdo tigrinus* named by Müller & Henle, 1837), the Lamniform *Squalicorax pristodontus* (Balbino & Antunes, 2007), Rajiformes (Delpiani, Figueroa & Mabragaña, 2012), and many other species within Carcharhiniformes and Lamniformes (Agassiz, 1843; Balbino & Antunes, 2007; Becker, Chamberlain & Stoffer, 2000; Boessenecker, 2016; Cappetta & Case, 1975; Davis, 1890; Gudger, 1937; Hubbell, 1996; Itano, 2013; Roemer, 1849; Shimada, 1997; Vuuren et al., 2015). Various pathologies have been reported in *O*. *megalodon* specifically (*e.g*., Renz, 2002); however, these specimens are not housed in public repositories.

We find multiple features point to gemination and/or fusion as the most likely etiology for the specimens of *O. megalodon* and *C. leucas* described herein including (1) a single incompletely split crown (as opposed to two crowns united by dentine and/or enameloid); (2) bifurcated, partially doubled internal morphology (pulp cavity in *C*. *leucas* and ascending canals of *O*. *megalodon*); and (3) only minor abnormalities to the root morphology along the midline. The latter includes a midline ridge with parallel grooves instead of a single midline groove, mesiodistally expanded nutrient groove near the central foramen, an internally bifurcating central foramen, and the possible presence of multiple central foramina in the pathological *C. leucas* tooth; and a concavity across the lingual surface between the neck and the crown in the pathological *O. megalodon* tooth.

There are several main issues that complicate etiological diagnosis of double tooth pathologies in sharks and other vertebrates, which prevents comparison with those of mammals. First is the absence of shared terminology reflective of shared etiology. Whereas double tooth pathologies in sharks are often referred to as “bicuspid” or “coalescent” (*e.g*., Balbino & Antunes, 2007; Botella, 2006; Botella, Valenzuela-Ríos & Martínez-Pérez, 2009; Delpiani, Figueroa & Mabragaña, 2012; Gudger, 1937), the terms “fusion,” “concrescence,” “gemination,” and/or “twinning” are common in mammalian studies. The latter terms are associated with developmental processes allowing direct etiological comparisons, whereas the former are generally ambiguous (undefined developmentally) with respect to splitting or fusion of the tooth bud during morphogenesis.

Mammals exhibit thecodont and largely diphyodont dentition (*i.e*., their teeth are completely enclosed in a deep socket of bone and most species only have two generations of teeth during their lifespan) (García & Zurriaguz, 2016). The teeth of sharks are not housed in sockets, rather they are attached to the top of mineralized cartilaginous jaws *via* connective tissues (acrodont) and infinitely regenerated throughout life (polyphyodont) (Ripamonti, 2018). Differences in tooth attachment, replacement frequency, and mode might affect how these double tooth pathologies manifest anatomically, or whether or not they occur at all. For example, incomplete breakdown of the dental lamina (a fold of oral epithelium that forms the tooth bud) is hypothesized to cause oral pathologies in mammals (Eversole, 1999; Štembírek et al., 2010), but is an irrelevant etiology in sharks due to the presence of a continuously erupting dental lamina in the latter (Ripamonti, 2018) (although laminar injury in sharks would be expected to cause tooth pathology). Disease and genetic mutations have been proposed as alternative possible causes of tooth deformities in mammals. Although there have been no functional studies evaluating the impact of disease and genetic mutations on dental development in sharks, these etiologies have largely been discounted in part due to modern selachians being particularly resistant to infections (Becker, Chamberlain & Stoffer, 2000 and references therein). Rather, tooth deformities in extant selachians, such as those resulting in tooth doubling, are generally hypothesized to be the result of trauma (Becker, Chamberlain & Stoffer, 2000; Gudger, 1937). Despite enormous variation in the form and function of vertebrate dentition, the structure and morphogenesis of teeth, the tooth regeneration process itself, and the genetic underpinnings of both are thought to be highly conserved throughout vertebrate evolution (Tucker & Fraser, 2014; Ripamonti, 2018). This suggests that developmental characterizations of tooth pathologies are likely to be homologous across vertebrates, and therefore the terminology proposed for mammalian double tooth pathology subtypes is likely to be widely applicable beyond mammals.

Second, there exists inconsistency in the application of mammalian terms for double tooth pathologies in the published literature, whereby the same terms are applied to different (hypothesized) developmental conditions and/or contrasting conditions are noted as key features for the same diagnosis. If terms and definitions lack standardization within mammals, this further complicates their extension outside the clade. For example, Kirillova (2009) describes tooth abnormalities in *Mammuthus* argued to represent damage to a single tooth bud during tooth morphogenesis as “fused” teeth. Based on the developmental condition, this etiology should equate to gemination. Hülsmann, Bahr & Grohmann (1997) describe human tooth abnormalities using the terms fusion and concrescence interchangeably when some examples should only equate to fusion and others should only equate to concrescence. Venkatesh et al. (2016) states that two distinct pulp cavities is a diagnostic feature of fusion, whereas a single pulp cavity is a diagnostic feature of gemination and cites Sekerci et al. (2011) for this definition. By contrast, Camargo, Aritaa & Watanabe (2016) state that fusion can be present with only one pulp cavity, even resulting in a single abnormally large tooth (More & Tailor, 2012). Finally, More & Tailor (2012) suggest that gemination, twinning, concrescence, and other terms are types of “fusion”, conflating all of these conditions with true fusion.

Perhaps the most serious impediments to comparing double tooth abnormalities during the evolution of vertebrates rests in the nature of the fossil record itself including the commonality of tooth preservation outside the jaw and the inability to directly observe tooth morphogenesis. This is because the diagnostic difference between fusion and gemination is a developmental one and it is therefore difficult to discriminate among these pathologies in isolated teeth (More & Tailor, 2012; Patil et al., 2013). Fusion is defined as the joining of two tooth buds in development, whereas gemination is defined as the interrupted splitting of a tooth bud resulting in a partially bifurcated tooth. Fusion can be complete, resulting in a single hypertrophied tooth, or incomplete resulting in separate crowns stemming from a single root, separate roots attached to a single crown, a partially divided root and single crown, or a partially divided crown and single root (Bhargava, Chaudhary & Aggarwal, 2012; More & Tailor, 2012; Patil et al., 2013; Camargo, Aritaa & Watanabe, 2016; Venkatesh et al., 2016). Any incomplete form of fusion can be morphologically indistinguishable from gemination, particularly when attempting to compare key diagnostic features noted for mammals to double tooth pathologies in sharks. For example in a study on human dentition, Kelly (1978) noted that because gemination is the splitting of a single tooth bud, the halves are predicted to be mirror images; whereas fusion typically represents fusion of a supranumerary tooth (typically abnormal in shape) to a normal tooth. However, in clear instances of gemination in the tiger shark *Galeocerdo cuvier* and the blacktip shark *Carcharhinus limbatus* caused by the puncture of a tooth germ by a stingray spine (Gudger, 1937; rediagnosed here in Table 2), the two halves of the pathological teeth are morphologically distinct, reflecting the original asymmetry of the non-pathological tooth structure. Therefore, this criterion may only apply to teeth that are normally symmetrical and cannot be generally applied. In addition, Kelly (1978) notes that fusion in mammals is typically characterized by the presence of two distinct roots, whereas in cases of gemination, there is usually only a single root. However, these frequency data are based on human studies and cannot confidently be applied widely across vertebrates without additional research and Kelly (1978) himself notes a seemingly conflicting example of this pattern. Given the difference in tooth implantation and root morphology between mammals and sharks, the diagnostic utility of such a feature outside mammals is questionable.

**Table 2:** Dental pathologies across Chondrichthyes.

Species name	Original identification of pathology	Pathology category (Fig. 7)	Source
*Carcharhinus leucas*	Double Tooth Pathology indet.	Double Tooth Pathology	This article
	Bent Cusp	Deformation	Becker, Chamberlain & Stoffer (2000)
*Carcharhinus obscurus*	Irregular series of tiny bud-like teeth lacking a central cusp	Irregularly Sized Tooth	Becker, Chamberlain & Stoffer (2000)
	Broken cutting edge due to teleost spine puncture	Deformation	Becker, Chamberlain & Stoffer (2000)
	Tooth Division/Split	Double Tooth Pathology indet.	Gudger (1937)
	Entire tooth bent backwards	Deformation	Gudger (1937)
*Carcharhinus plumbeus*	Broken cusps due to teleost spine puncture	Deformation/bending/twisting	Becker, Chamberlain & Stoffer (2000)
*Negaprion eurybathrodon*	Twisted crown	Deformation/bending/twisting	Balbino & Antunes (2007)
	Notched cutting edge	Cutting Edge Deformation	Balbino & Antunes (2007)
	Asymmetrically-shaped root	Root Deformation	Balbino & Antunes (2007)
*Carcharhinus sp*.	Tooth Division/Split due to internal division of tooth bud	Double Tooth Pathology indet.	Gudger (1937)
	Abnormally sized molar teeth	Irregularly Sized Tooth	Gudger (1937)
	Abnormal amount of teeth in tooth row	Irregular Tooth Row	Gudger (1937)
*Carcharhinus limbatus*	Tooth Division due to embedded sting-ray spine	Gemination	Gudger (1937)
*Galeocerdo cuvier*	Tooth Division due to embedded sting-ray spine	Gemination	Gudger (1937)
	Abnormally bicuspid tooth	Double Tooth Pathology indet.	Gudger (1937)
	Bent cusp	Deformation/bending/twisting	Gudger (1937)
	Cusp is reverse oriented mesially	Irregular Tooth Row	Balbino & Antunes (2007)
	Bent cusp	Deformation/bending/twisting	Balbino & Antunes (2007)
*Carcharodon*	Hooked cusp	Deformation/bending/twisting	Hubbell (1996)
	Tooth separated into two distinct teeth due to damaged gum tissue	Double Tooth pathology indet.	Hubbell (1996)
	Perforated tooth due to sting-ray spine	Perforation	Hubbell (1996)
	Two adjacent teeth merged together	Double Tooth Pathology indet.	Hubbell (1996)
	Entire tooth twisted	Deformation/bending/twisting	Hubbell (1996)
	Extreme deformation of tooth	Deformation/bending/twisting	Hubbell (1996)
*Paranomotodon sp*.	Cusp rotation	Deformation/bending/twisting	Becker, Chamberlain & Stoffer (2000)
	Deformed nutrient grooves	Nutrient Groove Deformation	Becker, Chamberlain & Stoffer (2000)
	Twisted cusp	Deformation/bending/twisting	Cappetta & Case (1975)
*Carcharoides totuserratus*	Cusp is reverse oriented mesially	Irregular Tooth Row	Balbino & Antunes (2007)
	Bent cusp	Deformation/bending/twisting	Balbino & Antunes (2007)
	Twisted cusp	Deformation/bending/twisting	Balbino & Antunes (2007)
	Abraided cutting edge, devoid of denticles	Cutting Edge Deformation	Balbino & Antunes, 2007
	Entire tooth twisted	Deformation/bending/twisting	Balbino & Antunes, 2007
*Carcharias taurus*	Twisted cusps	Deformation/bending/twisting	Vuuren et al. (2015)
	Notched cutting edge	Cutting Edge Deformation	Vuuren et al. (2015)
	Reduced cusplet size	Irregularly Sized Tooth	Vuuren et al. (2015)
*Scapanorhynchus texanus*	Bent Cusp	Deformation/bending/twisting	Becker, Chamberlain & Stoffer (2000), Roemer (1849)
	Abnormal root growths	Root Deformation	Roemer (1849)
*Archaeolamna kopingensis*	Rotated and compressed cusp	Deformation/bending/twisting	Becker, Chamberlain & Stoffer (2000)
	Entire tooth and crown bent	Deformation/bending/twisting	Davis (1890)
*Cretoxyrhina mantelli*	Notched cutting edge	Cutting Edge Deformation	Shimada (1997)
	Enameloid cracking	Enameloid Deformation	Shimada (1997)
	Excess growth of dentine	Irregularly Sized Tooth	Shimada (1997)
	Formation of fossae	Formation of Fossae	Shimada (1997)
	Protuberances on crown surface	Enameloid Deformation	Shimada (1997)
	Disturbance near cown-root contact	Neck Deformation	Shimada (1997)
*Otodus megalodon*	Double Tooth Pathology indet.	Double Tooth Pathology indet.	This article
	Split cutting edge	Cutting Edge Deformation	Itano (2013)
	Wavy cut in cutting edge	Cutting Edge Deformation	Boessenecker (2016)
	Lack of enameloid near base of crown and cutting edge	Enameloid Deformation	Balbino & Antunes (2007)
	Asymmetrically-shaped crown curving distally	Deformation/bending/twisting	Balbino & Antunes (2007)
*Squalicorax kaupi*	Distal notch disconnected	Cutting Edge Deformation	Agassiz (1843)
*Squalicorax pristodontus*	Bending along mesial edge	Deformation/bending/twisting	Agassiz (1843)
	Coalescent teeth	Double Tooth Pathology indet.	Balbino & Antunes (2007)
*Heterodontus portusjacksoni*	Abnormally sized molar teeth	Irregularly Sized Tooth	Gudger (1937)
	Abnormal amount of teeth in tooth row	Irregular Tooth Row	Gudger (1937)
*Chlamydoselachus anguineus*	Excess amount of cusps	Double Tooth Pathology indet.	Gudger (1937)
	Abnormally small teeth	Irregularly Sized Tooth	Gudger (1937)
	Double Teeth/Twinning	Double Tooth Pathology indet.	Gudger (1937)
*Amblyraja doellojuradoi*	An additional incomplete tooth row between two complete rows	Irregular Tooth Row	Delpiani, Figueroa & Mabragaña (2012)
	An increasing tooth base size and division of cusps	Double Tooth Pathology indet.	Delpiani, Figueroa & Mabragaña (2012)
	Irregular tooth arrangement	Irregular Tooth Row	Delpiani, Figueroa & Mabragaña (2012)
	Underdeveloped cusps, abnormally sized	Irregularly Sized Tooth	Delpiani, Figueroa & Mabragaña (2012)
	Deformed base/root	Root Deformation	Delpiani, Figueroa & Mabragaña (2012)
*Brachyrhizodus wichitaensis*	S-shaped tooth deformation	Deformation/bending/twisting	Becker, Chamberlain & Stoffer (2000)
	Offset nutrient grooves	Nutrient Groove Deformation	Becker, Chamberlain & Stoffer (2000)
	Entire tooth twisted	Deformation/bending/twisting	Romer (1942)
*Mobula rochebrunei*	Double Tooth Pathology indet.	Double Tooth Pathology indet.	Herman et al. (2000)
	Tooth row splitting	Irregular Tooth Row	Underwood & Cumbaa (2010), Herman et al. (2000)
*Cretomanta canadensis*	Pathologic fused teeth	Fusion	Underwood & Cumbaa (2010)
*Leonodus carlsi*	Pathologic fused teeth	Fusion	Botella (2006), Botella, Valenzuela-Ríos & Martínez-Pérez (2009)

**Note:**

Distribution of dental pathologies across Chondrichthyes corresponding with Fig. 7.

Several researchers have noted that the two pathologies can be differentiated by counting the number of teeth in the tooth row to determine if the total number of teeth is less or more than expected (*e.g*., Kelly, 1978; Patil et al., 2013). Other than direct observation of tooth morphogenesis, this is the only criterion we are aware of that has been published as a definitive means to discriminate between gemination and fusion of teeth. Unfortunately, this criterion cannot be evaluated in isolated teeth, which are common in the fossil record, especially for sharks.

Other diagnostic features may prove useful. Botella (2006), Botella, Valenzuela-Ríos & Martínez-Pérez (2009) identified an instance of fusion in isolated shark teeth based on the conjoining of two different-sized teeth in *Leonodus carlsi* (62% difference), indicating the two teeth were in different stages of morphogenesis at the time of fusion and thus revealing a developmental signal. We suggest that differences in the developmental stages (useful for sharks or mammals) or differences in crown morphology due to the fusion of different, yet neighboring, tooth types in heterodont taxa or aberrant supranumerary teeth (useful for mammals) are additional criteria that may prove to be reliable for differentiating between fusion from gemination in isolated teeth. Research on the efficacy for these traits to serve as criteria for etiological-based diagnosis of double tooth morphologies in extant sharks and other non-mammalian vertebrates is needed to make confident diagnoses.

The presence of internal bifurcation (main ascending canals in *O*. *megalodon*, and pulp cavity in *C*. *leucas*), only minor root abnormalities and a lack of clear root doubling, symmetrical mirroring of tooth halves (absence of any aberrant crown morphology, or developmental differences) suggests that gemination is more likely to have caused these double tooth pathologies; however, none of these features can be used to definitely rule out fusion for these specimens.

### Paleoecological inferences

To our knowledge, these are the first double tooth pathologies documented for either *O. megalodon* or *C. leucas* specifically; however, multiple other tooth pathologies are described and appear to be widespread in these taxa. Becker, Chamberlain & Stoffer (2000) document a pathologic *C. leucas* tooth in which the cusp of the tooth is bent forward towards the jaw symphysis. Pathologies in *O*. *megalodon* are abundant and include split cutting edges (Itano, 2013) among other abnormalities. These include teeth bearing subtle, wavy cuts along the midpoint of the crown on the distal cutting edge (Boessenecker, 2016) and trauma to tooth germs that caused the teeth to buckle lingually and distally or caused distortion of the distal cutting edge (Purdy et al., 2001). In extant taxa such tiger sharks (*Galeocerdo cuvier*) and blacktip sharks (*Carcharhinus limbatus*), which have diets that consist of Batoidea (rays and skates), tooth abnormalities have been linked to feeding trauma, such as puncture by stingray spine (Gudger, 1937). Injury due to the perforation of teleost or selachian fish spines during feeding has also been suggested to initiate tooth deformities (Becker, Chamberlain & Stoffer, 2000). Therefore, the presence of gemination and/or fusion provides further support for feeding trauma in *C*. *leucas* and *O*. *megalodon*.

*C. leucas* is known to be a generalist predator, having a diet composed of a wide diversity of prey including taxa known to cause feeding-related traumas that have previously been hypothesized to be linked to observed tooth deformations in other sharks. These include rays, sawfish, other sharks, bony fish, and sea urchins, all of which could potentially inflict damage to a developing tooth bud (Estupiñán-Montaño et al., 2017).

The diet of *O. megalodon* has been inferred to largely consist of cetaceans and sirenians based on general tooth morphology, estimations of bite force, predation and/or scavenging marks on prey, mechanical tooth damage, and evolutionary models (Godfrey et al., 2018; Diedrich, 2013; Medina-Gavilán et al., 2015 and references therein). It has also been suggested that *O. megalodon* fed on turtles and fish (Aguilera & de Aguilera, 2004). Identification of tooth abnormalities in *O. megalodon* resulting from damage or perforation of a developing tooth bud may provide support for a diet consisting of a wider diversity of marine animals. Although purely speculative, these could include spiny fish, billfish, walrus, and rays among other taxa. *Makaira* (marlin) is a genus of Istiophoridae (billfish) characterized by a distinctive spear-like rostrum used for hunting (Domenici et al., 2014). *Makaira* make up the diet of many modern sharks (Kitchell et al., 2002; Lowe et al., 1996) and interactions between these animals are known to occasionally become violent (Wisner, 1958), and can result in the wounding or death of the shark (Block, Booth & Carey, 1992). *Maikaira* and large spiny fish such as *Mola* (sunfish) are known to have inhabited the same environments as *O. megalodon* (*e.g*., the lower Middle Miocene Calvert Formation of Virginia (Weems, 1985; Carnevale & Godfrey, 2018; Perez et al., 2018)) and could have been food sources. In addition, the late Miocene Gatun Formation of Panama preserves a paleonursery habitat for *O. megalodon* and also a high diversity of selachians including *C. leucas* (Pimiento et al., 2013). Batoids (rays and skates) are also abundant in this formation and species such as *Aetobatus* (eagle rays), which are known to inhabit open waters and coral reefs, possess venomous tail barbs that are used defensively (Caceres et al., 2020; Schluessel, Bennett & Collin, 2010).

The late Neogene Purisima Formation of Northern California preserves a nearshore and estuarine environment and is represented by a highly diverse aquatic and terrestrial fauna including sharks such *O. megalodon*, rays, bony fish, toothed and baleen whales, sirenians, and seals (Boessenecker, Perry & Schmitt, 2014). Among the pinnipeds from the Purisima Formation, *Valenictus*, an extinct genus of Odobenidae (walrus), had tusks that likely grew to be nearly half a meter long (Boessenecker, 2017; Deméré, 1994). The Greenland shark *Somniosus microcephalus* likely feeds upon modern walruses (MacNeil et al., 2012), and a similar predator-prey relationship may have existed between *O. megalodon* and *Valenictus*. The tusks of walruses are more blunt than fish spines and ray barbs. Nonetheless, they are capable of inflicting serious injury upon the polar bears that hunt them (Ovsyanikov, 1995). If *Valenictus* constituted a portion of the diet of sharks in the Purisima fauna, their tusks may have posed a puncture risk to the developing tooth buds of *O. megalodon*.

Finally, interactions between conspecifics or consexuals are well documented in elasmobranchs (Martin, 2007; Brunnschweiler & Pratt, 2008), as is cannibalism (Gudger, 1932; Vorenberg, 1962; Budker, 1971; Snelson, Mulligan & Williams, 1984; Wetherbee, Gruber & Cortés, 1990; Vögler, Milessi & Quiñones, 2003). Interactions such as these may occasionally involve mouth to mouth biting between individuals, potentially resulting in damage to the tooth bud and subsequent deformation of teeth, and could also be a source of oral trauma. Although we consider these abnormalities in *C*. *leucas* and *O. megalodon* to most likely be the result of feeding trauma, it is clear that in some cases of abnormal tooth doubling in selachians, such as the enigmatic shark *Cretomanta*, trauma due to feeding is unlikely, as this shark was presumably planktivorous (Underwood & Cumbaa, 2010).

## Conclusions

We describe the internal and external morphology of pathological and non-pathological teeth of the lamniform *Otodus megalodon* and carcharhiniform *Carcharhinus leucas*, including the first three-dimensional reconstructions of the internal microstructure of the teeth of these taxa. Our pathological teeth exhibit a single bifid crown with symmetrical halves and abnormal internal microstructure including a bifurcating pulp cavity in *C. leucas* and more than twice as many main ascending canals in *O. megalodon*. We rediagnose the double tooth pathologies in *Galeocerdo cuvier* and *Carcharhinus limbatus* as gemination based on puncture of a tooth germ by a stingray spine, which yields a developmental signal; however, diagnosing the isolated *C*. *leucas* and *O*. *megalodon* teeth in our sample is more complicated. A bifurcating pulp cavity and a bifurcating main ascending canal in *C*. *leucas* and *O*. *megalodon* respectively, and the lack of major root abnormalities in both taxa, suggests gemination is a more likely diagnosis. This is supported by the symmetry of these teeth, which rules out fusion of tooth buds in one tooth file in different developmental stages, a criterion that has been used to diagnose the only instance of documented fusion in chondrichthyans (Botella, 2006; Botella, Valenzuela-Ríos & Martínez-Pérez, 2009). However, symmetry cannot be used to rule out fusion of a neighboring tooth in a single row in polyphyodont taxa. Therefore in the absence of total tooth count we opt for a more conservative diagnosis of gemination and/or fusion for these teeth.

Double tooth pathologies in sharks are largely hypothesized to stem from trauma to developing tooth buds. *C. leucas* is known to feed on a variety of prey documented to cause feeding-related traumas such as rays, sawfish, other sharks, bony fish, and sea urchins. The presence of double tooth pathologies in *O*. *megalodon* raises the question of whether the diet of this species (considered to consist mainly of marine mammals and possibly turtles and fish) was wider than currently appreciated. Additional study would be needed to link specific prey items to frequency of dental pathologies in sharks before confident dietary inferences could be made.

Terminology, differential diagnoses, and definitions of double tooth pathologies are often inconsistently applied to extant and fossil specimens including mammalian and non-mammalian species making comparisons difficult. We argue for a consistent set of definitions and diagnostic criteria that may permit a more detailed understanding of the evolutionary history and prevalence of various dental pathologies in Chondrichthyes and comparatively across vertebrates. Such an effort may lead to new associations with behavioral, dietary, or paleopathological factors such as disease and trauma that can increase our understanding of the paleobiology of ancient animals.

## Supplemental Information

10.7717/peerj.12775/supp-1 Supplemental Information 1 Vascularity measurements of *O. megalodon* teeth. Table shows diameter measurements of multiple main ascending and secondary canals in *O. megalodon* teeth (NCSM 9545 and NCSM 33639) at five different localities throughout the tooth from the root to the apex. The average diameter of the main ascending and secondary canals are calculated, as well as the difference in diameter between the two canal types for both teeth. A total average is calculated for the diameter of the main ascending and secondary canals and the difference in diameter between the two canal types. All measurements are taken in units of μm.

## Additional Information and Declarations

### Data Availability

The following information was supplied regarding data availability: Data is available at MorphoSource, all specimens are housed at the North Carolina Museum of Natural Sciences, accession numbers: NCSM 33639, NCSM 9545, NCSM 34038, NCSM 33640, NCSM 33641. 1) NCSM 9545 (Otodus megalodon): https://www.morphosource.org/concern/media/000368558?locale=en. 2) NCSM 33639 (Otodus megalodon): https://www.morphosource.org/concern/media/000368529?locale=en. 3) NCSM 29144, 33640, 33641 (Carcharhinus leucas): https://www.morphosource.org/concern/media/000368459?locale=en.
